
JOURNAL INFORMATION
==============================
NLM Title Abbreviation: Innov Aging
Journal ID: Innov Aging
NLM Journal ID: 101703706
Journal ID: innovateage

Innovation in Aging

EISSN: 2399-5300
Publisher: Oxford University Press

ARTICLE INFORMATION
==============================
PMCID: PMC10737085
DOI: 10.1093/geroni/igad104.2712
Article ID: igad104.2712
Article version: 1
Subjects: Abstracts; Session 7600 (Poster); Race, Ethnicity, Nativity, Culture, and Health; AcademicSubjects/SOC02600

DIMINISHED RETURNS ACROSS THE LIFE COURSE FOR BLACK INDIVIDUALS WITH TYPE 1 DIABETES

Bell Mallory Purdue University, West Lafayette, Indiana, United States

Publication date: 2023 Dec
Electronic publication date: 2023 Dec 21
Volume: 7
Issue: Suppl 1
Issue title: Program Abstracts from The GSA 2023 Annual Scientific Meeting, “Building Bridges > Catalyzing Research > Empowering All Ages”
First page: 841
Last page: 841
Copyright: © The Author(s) 2023. Published by Oxford University Press on behalf of The Gerontological Society of America.
Copyright year: 2023
License: This is an Open Access article distributed under the terms of the Creative Commons Attribution License (https://creativecommons.org/licenses/by/4.0/), which permits unrestricted reuse, distribution, and reproduction in any medium, provided the original work is properly cited.
License URL: https://creativecommons.org/licenses/by/4.0/

Page count: 1

==============================

### SESSION 7600 (POSTER)

### RACE, ETHNICITY, NATIVITY, CULTURE, AND HEALTH

Abstract citation ID: igad104.2712

**DIMINISHED RETURNS ACROSS THE LIFE COURSE FOR BLACK INDIVIDUALS WITH TYPE 1 DIABETES**

Mallory Bell, *Purdue University, West Lafayette, Indiana, United States*

Prior literature suggests that Black individuals of high socioeconomic status experience diminished returns on an array of health outcomes. However, few studies have examined whether the association between income and glycemic control varies by race among people with Type 1 diabetes (T1D) across the life course. In the current study, we extend prior literature by using a life course framework to examine the ways in which income interacts with race on glycemic control. Using data from the 2017-2018 wave of the T1D Exchange Registry (N=12,855), our results reveal that black individuals with T1D experience diminished returns on health: Black individuals with an annual income of $100,000 had worse glycemic control, operationalized by HbA1c level, compared to White individuals with an annual household income of $25,000. These racial disparities narrow among older adults. The current study suggests the need for a better understanding of the interpersonal and structural barriers associated with suboptimal glycemic control among Black individuals with T1D, especially as it relates to different stages across the life course.
